# Regulation of autophagy, lipid metabolism, and neurodegenerative pathology by heparan sulfate proteoglycans

**DOI:** 10.3389/fgene.2022.1012706

**Published:** 2023-01-09

**Authors:** Nicholas Schultheis, Robert Becker, Gelila Berhanu, Alexander Kapral, Matthew Roseman, Shalini Shah, Alyssa Connell, Scott Selleck

**Affiliations:** ^1^ Department of Biochemistry and Molecular Biology, Pennsylvania State University, University Park, PA, United States; ^2^ Department of Biochemistry and Microbiology, University of Victoria, Victoria, BC, Canada

**Keywords:** autophagy, lipid metabolism, mitochondria, heparan sulfate, proteoglycans, presenilin, nicastrin, co-receptors

## Abstract

Heparan sulfate modified proteins or proteoglycans (HSPGs) are an abundant class of cell surface and extracellular matrix molecules. They serve important co-receptor functions in the regulation of signaling as well as membrane trafficking. Many of these activities directly affect processes associated with neurodegeneration including uptake and export of Tau protein, disposition of Amyloid Precursor Protein-derived peptides, and regulation of autophagy. In this review we focus on the impact of HSPGs on autophagy, membrane trafficking, mitochondrial quality control and biogenesis, and lipid metabolism. Disruption of these processes are a hallmark of Alzheimer’s disease (AD) and there is evidence that altering heparan sulfate structure and function could counter AD-associated pathological processes. Compromising presenilin function in several systems has provided instructive models for understanding the molecular and cellular underpinnings of AD. Disrupting presenilin function produces a constellation of cellular deficits including accumulation of lipid, disruption of autophagosome to lysosome traffic and reduction in mitochondrial size and number. Inhibition of heparan sulfate biosynthesis has opposing effects on all these cellular phenotypes, increasing mitochondrial size, stimulating autophagy flux to lysosomes, and reducing the level of intracellular lipid. These findings suggest a potential mechanism for countering pathology found in AD and related disorders by altering heparan sulfate structure and influencing cellular processes disrupted broadly in neurodegenerative disease. Vertebrate and invertebrate model systems, where the cellular machinery of autophagy and lipid metabolism are conserved, continue to provide important translational guideposts for designing interventions that address the root cause of neurodegenerative pathology.

## 1 Introduction and background

### 1.1 Heparan sulfate biosynthesis and functions of heparan sulfate proteoglycans

**FIGURE 1:**
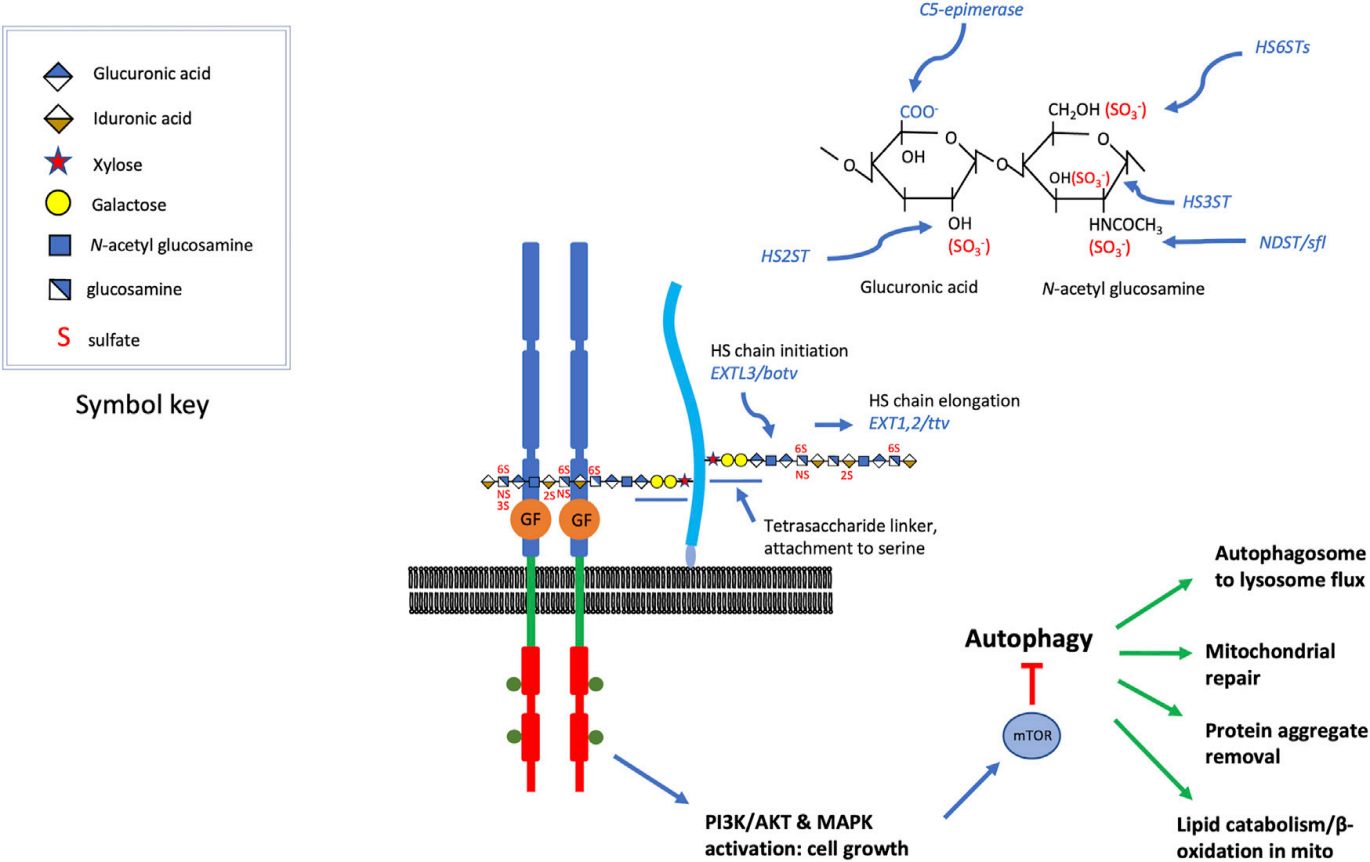
HSPGs serve as growth factor co-receptors. For a variety of secreted growth factors, HSPGs provide co-receptor function (Hassan et al., 2020), promoting the binding of the growth factor to the signaling receptor (the figure is roughly modeled on FGF, FGFR assembly) (syndecans are transmembrane and glypicans bear a GPI-linkage, which is depicted here). The heparan sulfate chains are critical for the assembly of these signaling complexes and some of the key genes affecting heparan sulfate modification are shown. These include, *O-*linked sulfotransferases, an epimerase, and an *N-*deacetylase *N-*sulfotransferase. Heparan sulfate chain initiation, added to the non-reducing end of a tetrasaccharide linker attached to specific serine residues of the core protein, requires the activity of the glycosyltransferase encoded by *EXTL3* or *brother of tout-velu (botv)* in humans and *Drosophila,* respectively. Chain elongation is achieved by the activity of enzymes encoded by *EXT1/2* or *tout velu (ttv)* and *sister of tout velu (sotv),* in humans and *Drosophila* respectively (non-reducing end toward polymer terminus). Signaling mediated by many of the growth factors affected by heparan sulfate co-receptors activate PI3 kinase or MAPK, inputs that regulate Target of Rapamycin (TOR) activity. These growth promoting functions also serve to suppress catabolic activity, in large measure *via* mTOR inhibition of autophagy. Some of the cellular functions of autophagy are listed, and these are suppressed when growth factor signaling is high. The elevation of autophagy and catabolic activity occurs when heparan sulfate biosynthesis is compromised, reducing the activity of the growth factors for which they serve as co-receptors.

Heparan sulfate proteoglycans (HSPGs) are an abundant class of cell surface and extracellular matrix proteins. Two widely expressed members of this protein class include syndecans and glypicans, integral membrane HSPGs represented by 4 syndecan (a transmembrane protein) and 6 glypican (GPI-linked proteins) homologs in vertebrates. Extracellular matrix or basement membrane localized HSPGs include Perlecan, Collagen type XVIII and Agrin. HSPGs typically bear two or three heparan sulfate chains covalently attached to specific serine residues of the protein core. The heparan sulfate modification is a linear polymer of alternating glucuronic acid and *N-*acetyl glucosamine sugars (Figure 1). Glucuronic acid can be epimerized to iduronate, and both sugars can be sulfated at various positions, reactions catalyzed by specific sulfotransferases (Figure 1). The biochemistry and enzymology of heparan sulfate biosynthesis has been reviewed in detail (Esko and Selleck, 2002; Merry et al., 2022) but the critical features for consideration here are: 1) the sulfation modifications are critical for interactions with protein ligands, 2) there is a great deal of structural heterogeneity with 3-*O,* 2-*O,* 6-*O* and *N-* residues that can bear sulfation modifications, 3) the pattern of sulfation along one chain is heterogeneous, with alternating patches of highly *versus* modestly sulfated regions, 4) the heparan sulfate chain is attached to the protein *via* a tetrasaccharide-linker that is shared with other glycosaminoglycans, such as chondroitin sulfate. It is critical to bear in mind that the pattern of sulfation is critical for function, and disturbances of that pattern have important consequences. In the course of our genetic studies of heparan sulfate biosynthetic enzyme encoding genes in *Drosophila* it became evident that *sulfateless (sfl),* the *Drosophila* homolog of the vertebrate gene encoding *N-*deacetylase *N-*sulfotransferase (*NDST1),* generally has the most dosage sensitive effects (Toyoda et al., 2000a; He et al., 2014; Weigelt et al., 2020) compared to a polymerase encoded by *tout velu (ttv*), the *Drosophila* homolog of vertebrate *EXT1*. This is likely a result of *N-*sulfation affecting the levels of subsequent *O-*sulfation at other positions and hence the regional pattern of sulfation along heparan sulfate chains. Reductions in heparan sulfate polymerase function affects chain length, but not sulfation state (Toyoda et al., 2000b; Okada et al., 2010).

### 1.2 Heparan sulfate proteoglycans as growth factor co-receptors

There has been an explosion of research during the last 25 years on the function of HSPGs at the molecular, cellular, and organismal level. Early pioneering work established that HSPGs are critical for FGF signaling (Olwin and Rapraeger, 1992; Aviezer et al., 1994), serving as co-receptors for assembly of an FGF-FGFR complex. Starting in the early 1990s, genetic studies of individual proteoglycans, such as the glypican homolog in *Drosophila, division abnormally delayed (dally)* (Nakato et al., 1995)or Glypican-3 in the mouse (Cano-Gauci et al., 1999), for example, demonstrated the broad effects of these molecules on growth and patterning events. Mutations compromising heparan sulfate biosynthesis show profound effects on signaling mediated by many secreted proteins that are instrumental in developmental patterning, including Wnt, Hedgehog and Bone Morphogenetic Proteins (BMP) family members (Lin et al., 1999; Bornemann et al., 2004). The list of growth factors regulated by heparan sulfate modified co-receptors is long indeed, and includes FGFs, Wnts, Hedgehogs, Hepatocyte Growth Factor, Heparin Binding-Epidermal Growth Factor, Platelet Derived Growth Factor, BMPs and many receptor tyrosine kinases (Hassan et al., 2020; Ohkawa et al., 2021). HSPGs also serve to control the stability and distributions of secreted protein factors in the matrix, providing another mechanism for regulating signaling (Takei et al., 2004; Nakato et al., 2016).

Many of the growth factors that are affected by heparan sulfate modified co-receptors signal *via* the phosphatidylinositol 3-kinase/Target of Rapamycin (PI3K/TOR) or ERK/MAPK pathways (Figure 1). These provide critical conduits for integrating growth and protein synthesis stimuli as well as the counteractive catabolic pathway of autophagy (Wang and Levine, 2010). Indeed, both PI3K and MAPK signaling can affect TOR activity, a suppressor of autophagy (Liu and Sabatini, 2020). Growth promoting signals are therefore coupled to autophagy inhibition, thus limiting catabolism when cells are receiving signals to grow and divide. Conversely, removal of growth stimuli provides a powerful means of activating autophagy, further demonstrating the counter-coupling of anabolic and catabolic processes. These signaling networks provide a conceptual framework for appreciating how compromising heparan sulfate biosynthesis, or reductions in the levels of HSPGs can produce activation of autophagy, as has been documented in both invertebrate and vertebrate model systems [reviewed in (Gubbiotti and Iozzo, 2015; Schultheis et al., 2021)].

One of the pathways modulated by heparan sulfate modified co-receptors, the MAPK/ERK pathway, also has profound effects on mitochondrial quality control and biogenesis. ERK1/2 activation regulates the balance between mitochondrial fission and fusion *via* suppressing Drp1 and promoting mitofusin activity, while repressing transcription of PGC1-α, a critical mitochondrial biogenesis signal (Collier et al., 2016; Chen et al., 2022). HSPGs participate in the activation of ERK *via* several growth factor inputs, including FGF. MAPK/ERK signaling thus provides a potential mechanism for HSPGs to modulate mitochondrial quality control and numbers, and thus cell metabolism.

### 1.3 Heparan sulfate proteoglycans as apolipoprotein receptors and their functional interaction with ApoE

HSPGs on the cell surface serve as receptors for several ligands, including apolipoprotein particles (Mahley and Ji, 1999; MacArthur et al., 2007; Gordts and Esko, 2018). Heparan sulfate modification mediated by NDST1 is critical for uptake of triglyceride rich lipoproteins (TRL) (MacArthur et al., 2007). In hepatocytes, this clearance is mediated by Syndecan-1 (SDC1) (Stanford et al., 2009) and provides an important mechanism independent of LDLR and LRP1 (Foley et al., 2013)-mediated internalization. ApoE and ApoAV are two lipoproteins that are important for SDC1 binding and uptake of TRLs (Gonzales et al., 2013). These findings establish that HSPG-ApoE interactions are biologically important.

The interaction between HSPGs and ApoE is relevant in the context of AD pathogenesis. It has been noted that different variants of ApoE that affect susceptibility to late onset AD show differences in heparin/heparan sulfate binding (heparin is a highly sulfated form of heparan sulfate), with disease-associated variants displaying the greatest affinity (ApoE4> ApoE3>ApoE2) (Arboleda-Velasquez et al., 2019). A recently described rare ApoE variant, ApoE3 Christchurch has greatly reduced binding to heparin and was associated with significant protection from early onset AD in one individual with *PSEN1*-mediated familial AD (Arboleda-Velasquez et al., 2019). Notably, a 70-year-old woman bearing a dominant *PSEN1* mutation and two copies of the ApoE3 Christchurch variant had only mild cognitive impairment, a delayed onset of 3 decades compared to cohorts bearing the *PSEN1* mutation alone. Functional MRI of this individual revealed high brain amyloid levels but remarkably reduced tau accumulation, showing that her relatively intact cognitive status correlated with low tau levels.

A recently published GWAS analysis identified two additional ApoE variants that also provide significant protection from late onset AD, APOE ε4 (R251G) and APOE ε3 (V236E) (Le Guen et al., 2022). Both variants reside in the carboxy domain of ApoE, a region shown to affect dimerization. More to the point of this discussion, the C-terminal segment of ApoE is important for heparan sulfate affinity, and ApoE dimerization appears to be critical for heparan sulfate binding (Yamauchi et al., 2008). The *APOE*3-V236E variant has indeed been shown to affect ApoE multimer formation (Liu et al., 2021). Further analysis of these new variants is needed to determine if the correlation between the heparan sulfate affinity and ApoE impact on AD applies to these ApoE forms as well. These findings raise the possibility that ApoE-heparan sulfate interactions influence the mechanism of ApoE-lipid uptake or lipid transport (Qi et al., 2021) and ultimately the mechanism of AD pathogenesis. It is also important to note the signaling functions of ApoE, including activation of Erk1/2. The disease associated variant, ApoE4 shows the greatest capacity to trigger phosphorylation and activation of Erk (Huang et al., 2019) and it is possible that a co-receptor function of HSPGs with ApoE is a determinant of their functional interplay in AD.

### 1.4 Effects of heparan sulfate proteoglycans on autophagy

There are many lines of evidence demonstrating that HSPGs normally serve to suppress autophagy, and hence compromising the synthesis or modification of heparan sulfate produces autophagy elevation (Schultheis et al., 2021). Mutations in key heparan sulfate biosynthesis enzyme-encoding genes increase both autophagosome and lysosome levels, and results in elevated flux through this membrane system in *Drosophila* Reynolds-Peterson et al., 2017 *.* These changes have physiological consequences as animals with reduced *sfl/Ndst1* activity for example, show increased resistance to oxidative stress, and an extended lifespan (Reynolds-Peterson et al., 2020). One of the signaling systems known to be affected by heparan sulfate co-receptor function, Ras-Erk, plays and important role in lifespan in *Drosophila* and inhibitors of MEK1/2, an upstream element of this pathway, extend lifespan (Slack et al., 2015). Reduction of Erk activation mediated by decreased co-receptor function may be the mechanism of altered *sfl* function affecting lifespan (Reynolds-Peterson et al., 2020; Weigelt et al., 2020).

Reduction of either *sfl/Ndst1* or *ttv/Ext1* also suppresses cellular abnormalities mediated by disruption of Presenilin or *Parkin/PARK2* in *Drosophila,* models or AD and Parkinson’s disease, respectively (Reynolds-Peterson et al., 2020). In the mouse, elevated autophagy is found in cells lacking the extracellular matrix proteoglycan, perlecan (Ning et al., 2015). Ectopic expression of heparanase, a heparan sulfate degradative enzyme, also activates autophagy broadly, supporting the model that HSPGs normally serve to suppress autophagy (Ilan et al., 2015; Shteingauz et al., 2015; Sanderson et al., 2017).

Further evidence that HSPGs suppress autophagy comes from the studies of disorders where heparan sulfate degradation is compromised. There disorders are collectively known as mucopolysaccharidoses, where mutations compromise the activity of specific lysosomal enzymes that degrade heparan sulfate (Heon-Roberts et al., 2020). These diseases are characterized by progressive neuronal degeneration and show a dramatic deficit in autophagy. MPS-IIIA for example, is caused by mutations in the gene encoding sulfaminidase, a lysosomal sulfatase, compromising stepwise degradation of heparan sulfate. Retinal degeneration in MPS-IIIA mice is accompanied by heparan sulfate accumulation, defective autophagy flux and reactive microgliosis (Intartaglia et al., 2020). A recent study reports a promising therapy of MPSIIIa using fluoxetine, a serotonin reuptake inhibitor that also activates TFEB, a transcriptional activator of lysosomal and autophagy functions (Capuozzo et al., 2022). *In vivo* treatment of MPS-IIIA mice with fluoxetine decreases glycosaminoglycan accumulation and aggregated autophagy substrates. All these studies emphasize that HSPGs are intimately involved in the regulation of catabolic processes, including autophagy.

## 2 Heparan sulfate proteoglycans in the context of Alzheimer’s disease

### 2.1 Discoveries that ushered in the modern era of AD research

To appreciate how HSPGs can affect Alzheimer’s Disease (AD) pathogenesis, a brief review of the history and current understanding of AD pathophysiology is warranted. The modern era of Alzheimer’s Disease (AD) truly began in 1984 with the identification of specific peptides as a principal component of neuritic plaques, an extracellular pathological hallmark of AD also described as amyloid (Glenner and Wong, 1984). In 1985 and 1986 a series of studies described the microtubule associate protein Tau as a component of paired-helical filaments, an intracellular pathology hallmark of AD known as neurofibrillary tangles. The relevance of Tau was supported by subsequent genetic studies showing mutations in Tau were associated with neurodegenerative disease (Goedert et al., 2012). Following close upon these discoveries was the identification of the gene that encoded amyloid-sequestered peptides, Amyloid Precursor Protein (APP) (Goldgaber et al., 1987; Kang et al., 1987; Tanzi et al., 1987). In 1991, a mutation in *APP* was found to segregate with familial and autosomal dominant early onset AD (Goate et al., 1991) and established that APP contributes to AD in a functionally significant manner. In 1994 genetic variants that confer either susceptibility or protection to late onset AD (LOAD) were found in *APOE,* a lipid binding protein critical for lipid transport and metabolism (Katzman, 1994). These findings implicated dysfunction in lipid metabolism as playing a major role in LOAD. By 1995 mutations in presenilin-1 and presenilin-2 were identified as causing autosomal dominant EOAD (Haass, 1996). Characterization of the enzymes encoded by *PSEN1* and *PSEN2* showed they were both components of a protease, γ-secretase, that cleaves APP into Aβ peptides. These discoveries set the stage for efforts to target amyloid generating processes for interventions that could hopefully block the development of AD. The clinical trial failures of an array of pharmaceuticals directed at reducing production or promoting removal of amyloid has prompted reevaluation of the amyloid hypothesis and whether amyloid deposits cause or are simply associated with AD pathology (Imbimbo and Watling, 2021). The failures of the pharmaceutical interventions, however, have not diminished the conclusion that APP processing, and presenilins are important in AD pathophysiology. Indeed, GWAS studies have strongly suggested and supported the involvement of APP processing in LOAD as well as the established contribution to EOAD (see section below). Recently, antibody or antisense drugs directed against Tau, another pathological feature of AD, have also been disappointing (Mullard, 2021). Why these approaches have failed is not fully understood but suggest the need for a more complete understanding of AD pathological mechanisms.

### 2.2 The evolving understanding of AD pathophysiology

**FIGURE 2:**
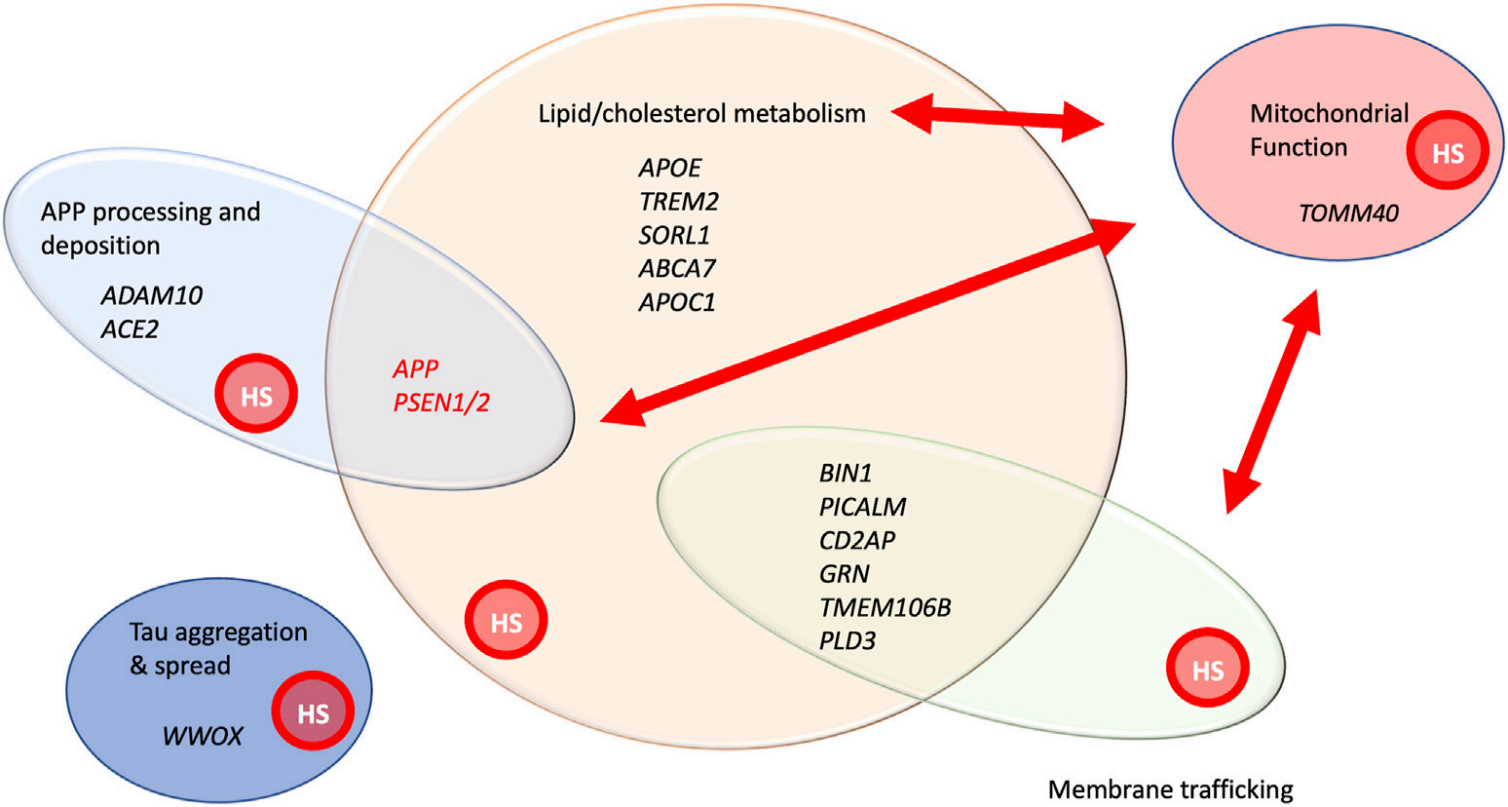
A functional snapshot of AD genetics and cellular processes implicated by genes involved in early onset, familial AD, as well genes implicated by GWAS analysis of late onset AD (Scheltens et al., 2021; Bellenguez et al., 2022). Genes in black print have been identified by either sequencing of rare variants or GWAS analysis. *APP* and *PSEN1/2* are shown in red print to highlight both their high penetrance in affecting AD and their association with early onset AD in families. Some of the genes with variants showing significant association with AD from GWAS are listed, representing functional categories. This is not intended to be a comprehensive list, but simply providing illustrative examples of genes within these groupings. Red arrows represent the important functional relationships between cellular processes and genes affecting them, with mitochondrial activity. The graphic overlay of groups represents that these are functionally connected groups and genes can participate in more than one of these processes. *All* these processes are known to be affected by HSPGs, denoted by the red circle labelled, “HS” within each functional group. References providing evidence for HSPG involvement in these processes include the following: APP processing and deposition (Snow et al., 1988; Jendresen et al., 2015; Liu et al., 2016), lipid/cholesterol metabolism (Yamashita et al., 2018), mitochondrial morphology and function (Reynolds-Peterson et al., 2017; Yamashita et al., 2018; Schultheis et al., 2021), membrane trafficking (Shteingauz et al., 2015; Reynolds-Peterson et al., 2017; Sanderson et al., 2017; Nadanaka and Kitagawa, 2018; Reynolds-Peterson et al., 2020) and Tau aggregation and spread.

**FIGURE 3:**
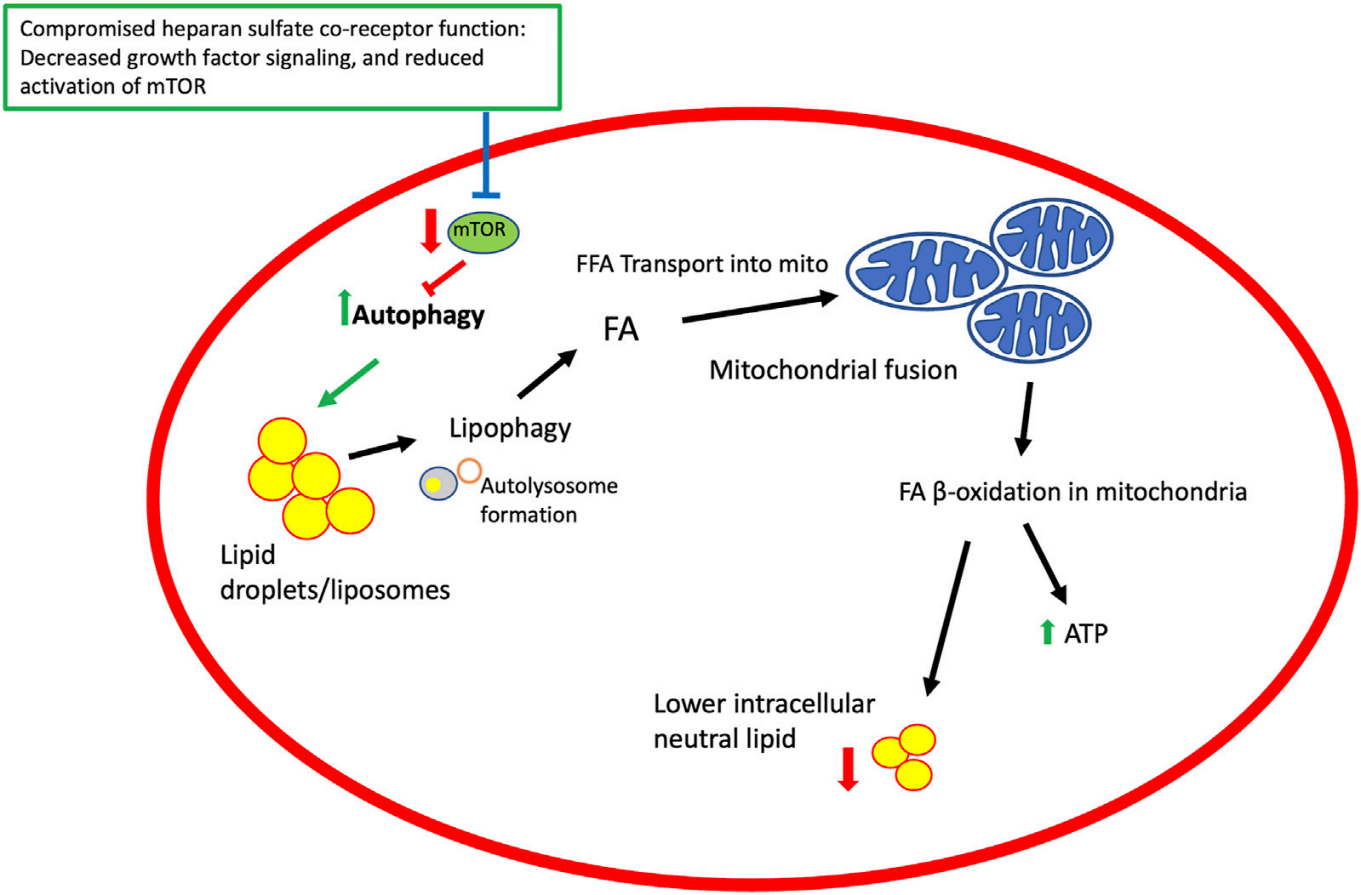
A model for heparan sulfate proteoglycan co-receptor function affecting autophagy, lipid metabolism and mitochondrial function. Growth factor signaling modulated by HSPGs affect signaling pathways that regulate mTOR activity. TOR is an inhibitor of autophagy and thus compromising mTOR activation promotes autophagy and downstream events, including lipophagy and mobilization of fatty acids from lipid droplets, mitochondrial fusion, and β-oxidation of lipids in mitochondria to generate ATP and lower intracellular lipid stores. Elements of this figure reflect events summarized in a published review (Hsu et al., 2021).

Over the last several years, genome wide association studies (GWAS) and whole exome sequencing have identified more than 70 genes that confer susceptibility to AD (Bellenguez et al., 2022) (Figures 2, 3). The advent of improved sequencing allowed the identification of rare variants that contribute substantial risk for AD, including *TREM2, SORL1* and *ABCA7*, genes that are also involved in lipid biology (Hoogmartens et al., 2021). Pathway enrichment analysis has highlighted the significant involvement of gene sets connected to APP, tau, lipids, endocytosis, and immune regulation (including macrophage and microglial cell function). A meta-analysis of genetic contributions to late onset AD (LOAD) identified lipid metabolism as one of four pathways selectively affected, with APP processing, immunity and tau-binding proteins completing the tetrad (Kunkle et al., 2019). These findings also emphasize that early and late onset mechanisms of AD are likely shared to a significant degree. Pathway and gene network analysis combining GWAS, and transcriptomics data revealed several interesting functional groups, including immune system regulation (particularly in microglia), lipid metabolism, membrane trafficking, and protein clearance and processing (Rosenthal et al., 2022). A large gene cluster centered around *APOE*, the most prevalent gene variant affecting LOAD, includes 49 genes, encoding other lipoproteins, *HS3ST*, a gene affecting heparan sulfate modification, and glypican-2 (*GPC2*) an integral membrane heparan sulfate modified protein. Another recent large GWAS study using MAGMA gene-set analysis identified 25 gene ontology biological processes enriched in LOAD-associated variants (Wightman et al., 2021), including 66 genes involved in lipid transport or metabolism. An overview of the cellular processes affected by variants or mutations in AD-associated genes is summarized in Figure 2.

It is important to point out that many of the AD-associated genes can readily be placed in more than one functional group (Figure 2). *TREM2* for example is primarily placed in the immunoregulatory group yet it has lipid ligands, namely APOE, and it mediates myelin phagocytosis, a membrane trafficking function. *APP*, the gene encoding the transmembrane protein clearly implicated in AD, and the source of amyloid accumulation, also affects lipid metabolism. Recent work shows that a 99 amino acid APP-*derived* peptide that serves as a substrate of γ-secretase in the ER influences *de novo* cholesterol synthesis and uptake into the plasma membrane (Montesinos et al., 2020). This overview of genetic data serves to emphasize that many of the pathways implicated in AD directly or indirectly affect lipid metabolism. Genes affecting membrane trafficking can also certainly influence lipid movement or disposition. APP-derived peptides affect cholesterol metabolism and *PSEN1/PSEN2* have effects on autophagosome to lysosome traffic, mitochondrial function, and lipid accumulation (Van Acker et al., 2019). *TOMM40,* a gene of the outer mitochondrial membrane, has been identified in GWAS analysis for association with AD (Brabec et al., 2021), and its expression is upregulated in *postmortem* AD brain (Lee et al., 2021). It is intriguing that the pathway analysis from large GWAS and exome sequencing efforts implicated several of the cellular processes disrupted in *PSEN1* mutant cells, suggesting that mechanistic insights gained from studies of *PSEN1* mutants may be broadly applicable to AD pathogenesis.

### 2.3 Heparan sulfate proteoglycans and their role in AD pathology

HSPGs have been implicated in AD pathology for over three decades. They were originally identified as components of extracellular amyloid aggregates, suggesting they could play a role in the stabilization, formation, or turnover of neuritic plaques, one of the pathological hallmarks of the disease (Snow et al., 1988; Celesia, 1991). Compromising heparan sulfate biosynthesis in mature neurons or ectopic expression of an enzyme that degrades heparan sulfate suppress the accumulation of amyloid in mouse models of AD (Jendresen et al., 2015; Liu et al., 2016). Later, it was discovered that HSPGs internalize Tau, affect Tau aggregation and release of misfolded Tau to neighboring cells, providing a mechanism of intercellular spread mediated by this protein (Holmes et al., 2013; Mah et al., 2021; Huynh et al., 2022; Song et al., 2022). HSPG-mediated uptake of monomeric Tau also activates Erk signaling, promoting pro-inflammatory processes (Song et al., 2022), another potential aspect of this heparan sulfate-mediated process. More recently HSPGs were found to regulate autophagy (Ning et al., 2015; Reynolds-Peterson et al., 2017; Reynolds-Peterson et al., 2020), and affect lipid accumulation (Yamashita et al., 2018), two processes that are also disrupted in AD. One of the principal genetic contributors to LOAD, APOE, has significant binding to HSPGs, implicating HSPGs in lipid transport and *APOE-*mediated AD disease processes. As described above, recent work discovered an *APOE3* variant bearing a mutation in the heparan sulfate binding domain that abrogated heparan sulfate affinity (Arboleda-Velasquez et al., 2019) and was associated with significant suppression of cognitive decline in an individual with a dominant *PSEN1* mutation that mediates familial, EOAD. The growing set of observations implicating heparan sulfate proteoglycans in neurodegenerative disease over the years has prompted a restatement and updating of the original proposal that HSPGs are critical for AD development (Snow et al., 2021). The emphasis of this review is on recent work demonstrating that HSPGs affect autophagy and membrane trafficking, as well as lipid metabolism, and the implications of these activities for AD pathogenesis. Further, we present a model system for studying the cellular function of presenilin and nicastrin, two components of the γ-secretase, in the fruit fly *Drosophila.*

## 3 Models for studying Alzheimer’s disease cellular and molecular pathology

### 3.1 AD-related pathophysiology in Drosophila; conservation of γ-secretase components, its substrates, and cellular functions

For some time, it was assumed that the dominant *PSEN1* mutations conferring susceptibility to early onset AD were gain- or altered-function alleles that resulted in increased Aβ production or a change in the balance of APP-derived Aβ40/42 peptides. Subsequent biochemical analysis of 138 pathogenic mutant forms of presenilin 1 in a reconstituted γ-secretase enzyme showed that 90% of these displayed reduced Aβ peptide production (Sun et al., 2017). This has important implications for considering how to model disruptions of presenilin 1 function in a manner that informs the disease state. In short, loss of function (LOF) or partial LOF mutants of *PSEN1* are relevant to known pathological mutations from AD patients. In the fruit fly *Drosophila,* homologs of all 4 protein components of vertebrate γ-secretase are represented, including Presenilin (Psn), the catalytic unit, as well as Nicastrin (Nct), an important regulatory component that influences substrate selection (Pamren et al., 2011). Reductions of *Psn* function in *Drosophila* leads to cell loss in the brain, and retina, even when gene function is compromised only in mature neurons of the adult animal (Kang et al., 2017). These findings argue that studying Presenilin function in *Drosophila* is relevant to determining how this molecule can affect the initiation and progression of AD-related pathogenesis. With regard to understanding the role of HSPGs in AD pathogenesis and their potential interaction with presenilin, the evolutionary conservation in *Drosophila* to vertebrate systems extends to the principle HSPGs (glypicans, syndecans, perlecan), as well as the complex machinery required to synthesize and modify heparan sulfate chains (Toyoda et al., 2000a).

Presenilin 1, in addition to providing the protease activity that generates amyloid-producing peptides, has important cellular functions. This is particularly relevant since the direct role of amyloid deposition in AD pathological mechanisms is in question and other functions of presenilin may therefore be critical to the development of AD. Reductions of presenilin function or expression of Presenilin mutant proteins derived from patients, show disruptions in mitochondrial function, autophagy to lysosome trafficking, and lipid metabolism (Deaton and Johnson, 2020). It is important to point out that pathway analysis of GWAS variants associated with LOAD has identified membrane trafficking, APP and Tau processing, membrane trafficking and lipid metabolism as over-represented functional domains, indicating these processes may be critical to the pathological and mechanistic events behind all forms of AD (Kunkle et al., 2019). These processes are also functionally connected, for example, membrane trafficking events are critical for mitochondrial function and lipid catabolism.

Mitochondria play a central role in lipid catabolism and changes in their morphology accompany activation of lipid breakdown. Mitochondria serve the critical function of β-oxidation of fatty acids to generate ATP through the Kreb’s cycle and import of fatty acids into the mitochondrial matrix is an essential element of lipid catabolism. Cell starvation and activation of macroautophagy results in elevated lipid delivery to mitochondria, and net oxidation of fatty acids from liposome stores to produce ATP. Mitochondrial fusion occurs upon autophagy activation (Gomes et al., 2011), providing a mitochondrial network for efficient fatty acid distribution. Inhibition of mitochondrial fusion by knockdown of Mitofusin in mouse embryonic fibroblasts impairs β-oxidation efficiency and promotes lipid export to neighboring cells (Rambold et al., 2015; Cohen et al., 2018). Central to the point, however, is that activation of autophagy and lipid catabolism is associated with dramatic changes in mitochondrial morphology because of fusion and reduction in liposome stores on account of elevated lipid catabolism. Conversely, inhibition of lipid catabolism and suppression of autophagy would be expected to produce small mitochondria, and an increased level of lipid in liposomes. Indeed, mutations in presenilin-1 produce small, dysfunctional mitochondria, and produce an accumulation of lipid (Sarasija et al., 2018; Rojas-Charry et al., 2020; Han et al., 2021).

We have begun an analysis of the cellular processes disrupted by reduction of presenilin or nicastrin function in the fruit fly *Drosophila melanogaster.* RNA interfering constructs have been generated for both genes, permitting cell and developmental timing-specific knockdown of their corresponding mRNAs (Kang et al., 2017). RNA interference of these two components of the γ-secretase enzyme have been shown to compromise Notch signaling and produce neuronal loss in both the brain and retina. Furthermore, neuron-directed knockdown of presenilin or nicastrin in adult animals leads to age-dependent behavioral deficits and neurodegeneration, demonstrating that the deficits are not developmental and that the function of these proteins are required continuously into adulthood (Kang et al., 2017).

Given the effects of presenilin mutations in vertebrate systems on mitochondrial function and lipid metabolism we have chosen to examine these processes in the principal metabolic regulatory organ of insects, the fat body. Fat body cells store both triglycerides and glycogen, releasing those energy reserves under conditions of high demand, such as the transition from larvae to the adult body plan that occurs during metamorphosis. Fat body cells provide functions served by both hepatocytes and adipocytes in vertebrate physiology. Experimentally they offer some definite advantages, namely their large size (60 μ) and ready access for confocal microscopy in both larvae and adult animals. It is also readily possible to knockdown genes selectively in fat body cells, using Gal4 bearing P-elements that express GAL4 in fat body, together with *UAS-GeneX* ^*RNAi*^ constructs (UAS is the binding site for GAL4). We have used *r4Gal4>UAS-shPsn* ^*RNAi*^ or *r4Gal4>UAS-shNct* ^*RNAi*^ to achieve knockdown of Presenilin or Nicastrin in fat body cells and evaluate the effects on mitochondrial number and morphology, autophagosomes, lysosomes, and liposomes, using both fluorescent markers for these compartments as well as transmission electron microscopy. Our preliminary findings indicate that compromising *presenilin* function in *Drosophila* has profound effects on mitochondria, autophagosome to lysosome traffic and lipid metabolism (data not shown), as has been documented for vertebrate systems. *Drosophila* therefore provides a powerful model to examine how presenilin and nicastrin deficits can lead to conserved cell pathology and what pathways can counter these deficits.

Important insights have been gained from studies of presenilin in the nematode, *C. elegans* (Sarasija and Norman, 2015; Ryan et al., 2021)*.* The homolog of presenilin in this roundworm is encoded by *sel-12,* that like its vertebrate counterparts, exhibits protease activity. Disruption of *sel-12* function produces mitochondrial fragmentation and disruption of Ca^++^ homeostasis. Furthermore, this role in Ca^++^-release from ER is independent of γ-secretase protease activity. Autophagy to lysosome trafficking is also suppressed in these mutants, apparently the result of mTOR hyperactivation. The broad conservation of signaling components and cellular pathology associated with presenilin deficits argue for the power of these simple model systems in a detailed understanding of early events that initiate and produce neurological compromise in AD patients.

### 3.2 Heparan sulfate biosynthesis affects mitochondrial morphology, autophagy flux and lipid metabolism in both *Drosophila* and vertebrate models

We have previously noted changes in mitochondrial morphology in *Drosophila* muscle cells upon activation of autophagy mediated by inhibition of key heparan sulfate biosynthetic steps (Reynolds-Peterson et al., 2017; Schultheis et al., 2021). Inhibition of two key heparan sulfate biosynthetic enzyme encoding genes, *ttv/Ext1* or *sfl/Ndst1* produced enlargement of mitochondria, suggesting a change in the fusion-fission balance. These morphological changes were suppressed by reducing the function of *Atg* genes, demonstrating these events were dependent on elevated autophagy that occurs because of compromised heparan sulfate biosynthesis. The effects of structural changes in heparan sulfate on mitochondrial morphology should be considered in the context of altered autophagy, which has an established impact on mitochondria and lipid metabolism.

Analysis of mouse perlecan (Hspg2) mutants have greatly informed our understanding of how a heparan sulfate modified protein regulates these processes. First, loss of perlecan in muscle can activate autophagy. Evidence suggests that this regulation of autophagy occurs *via* perlecan-mediated activation of the mTORC1 pathway (Ning et al., 2015). These findings are entirely consistent with other findings from mouse and *Drosophila* work, showing that heparan sulfate modified molecules serve to suppress autophagy. Recent work examined the function of perlecan/Hspg2 in liver, skeletal muscle, and adipocytes, demonstrated striking effects on mitochondrial function, and lipid metabolism (Yamashita et al., 2018). White adipose tissue mass was reduced upon removal of perlecan function with an accompanying reduction in adipocyte size. Liver cells showed lower levels of lipid accumulation under high fat diet conditions, and whole animal measures of metabolism demonstrated elevated fat oxidation in animals with compromised perlecan function. All these findings demonstrate that compromising the function of a single heparan sulfate modified protein of the extracellular matrix can increase lipid catabolism and increase mitochondrial number in skeletal muscle. The changes in mitochondrial density in muscle was associated with elevated levels of peroxisome proliferator-activated receptor gamma coactivator 1-alpha, an inducer of mitochondrial biogenesis.

Earlier work has shown that reducing the function of key heparan sulfate biosynthetic enzyme encoding genes increases autophagy flux, with elevation of both autophagosome and lysosome markers in both muscle and fat body. This change in autophagy has the capacity to rescue cell death in two contexts, dysfunction of presenilin expressed in the retina, and mutations in *parkin* that produce degeneration of flight muscle cells (Reynolds-Peterson et al., 2020). Given the spectrum of cell processes affected by reductions in presenilin or nicastrin function, in particular the changes in mitochondrial structure and increased levels of lipids, we are examining if compromising heparan sulfate biosynthesis in fat body produces compensatory effects. Our findings thus far are consistent with earlier work; modulating heparan sulfate biosynthesis, and hence events regulated by heparan sulfate modified proteins, have opposing and compensatory effects on multiple phenotypes resulting from knockdown of presenilin (unpublished findings). Collectively, evidence from multiple model systems indicates that inhibition of heparan sulfate biosynthesis and modification can counter many of the cellular changes produced by reductions in presenilin function.

## Conclusion

HSPGs play important roles in several processes involved in neurodegenerative pathology, including growth factor signaling, endocytosis, interaction with ApoE, propagation of misfolded Tau, and disposition of APP-derived peptides. In addition, their newfound roles in autophagy, mitochondrial function and lipid catabolism indicates that suppressing HSPG function could provide multiple ways of ameliorating cellular pathology found in neurodegenerative disease. While the direct role of amyloid deposition, or soluble APP-derived fragments in disease is open to question, the importance of APP and presenilin remains well supported by an abundance of genetic data. Both GWAS findings of late onset disease, and cellular biology of cells with characterized mutations in APP or presenilin point to dysfunction of lipid metabolism, mitochondria, and autophagy as common elements of neurodegeneration. Immune regulation in microglia, and the interaction of neurons with both microglia and astrocytes are also important determinants in AD outcomes. These cellular and genetic processes are conserved in model organisms, including *Drosophila.* The capacity of these simpler organisms to reveal the biology in an intact animal provides a powerful tool to invigorate our fundamental understanding of a set of disorders that currently remain out of reach of any effective treatment. There is good evidence to suggest that targeting of heparan sulfate biosynthesis could counter several cellular pathological processes common to neurodegenerative disease.
